# Pharmacological Control of Receptor of Advanced Glycation End-Products and its Biological Effects in Psoriasis

**Published:** 2013-09

**Authors:** A. V. Mezentsev, S. A. Bruskin, A. G. Soboleva, V. V. Sobolev, E. S. Piruzian

**Affiliations:** Federal Non-profit Research Institute of Russian Academy of Sciences, N.I. Vavilov Institute of General Genetics, Moscow 119991, Russia

**Keywords:** AGE, JAK kinases, KIOM-79, psoriasis, RAGE, SAGE, tyrphostins

## Abstract

Receptor for advanced glycation end-products is implicated in a development of chronic inflammatory response. Aim of this paper is to provide a review on commercial and experimental medicines that can interfere with RAGE and signaling through RAGE. We searched three bibliographical databases (PubMed, Web of Science and MEDLINE) for the publications from 2005 to March 2012 and identified 5 major groups of agents that can interfere with RAGE biological effects. In the first part of this paper, we discuss AGE crosslink breakers. These chemicals destroy advanced glycation end products (AGEs) that are crosslinked to the extracellular matrix proteins and can interact with RAGE as ligands. Then, we describe two non-conventional agents SAGEs and KIOM-79 that abolish certain biological effects of RAGE and have a strong anti-inflammatory potential. In the third part, we evaluate the inhibitors of the signaling cascades that underlie RAGE. Particularly, we discuss two groups of kinase inhibitors tyrphostins and the inhibitors of JAK kinases. Considering RAGE as a potential master regulator of processes that are crucial for the pathogenesis of psoriasis, we propose that these medicins may help in controlling the disease by abolishing the chronic inflammation in skin lesions.

## INTRODUCTION

Psoriasis is a chronic skin condition, and the prevalence of this condition in Europe is approximately 2% (1). Psoriasis is less common in individuals of Asian or African background. Only 0.7% of African Americans and 0.4% of Chinese in China are diagnosed with psoriasis (2). In Russia, the number of individuals that suffer from the disease is above 3% (3).

Psoriasis is a disease with genetic predisposition. Children with at least one parent who is diagnosed with psoriasis have a higher risk of developing the disease themselves (4, 5). The most common form of psoriasis, Psoriasis vulgaris, is responsible for more than 90% of cases. Psoriasis vulgaris or plaque-type psoriasis is characterized by the presence of red scaly plaques that exhibit a silvery, flaky upper skin layer that may appear anywhere on the body. Generally the plaques vary in size and tend to merge with each other as the disease progresses.

Receptor for advanced glycation end products, RAGE plays a crucial role in the inflammatory response (6, 7). By interacting with multiple ligands, which can be either in a soluble form or crosslinked to the extracellular matrix, RAGE activates several signaling mechanisms and regulates gene expression *via* a group of well-characterized transcription factors, such as NFκB and AP1 (8-10). Knocking RAGE out in mice prevents a development of chronic inflammation (11).

The expression of RAGE in both immune cells and their targets (12-14), a high stability of RAGE in complexes with ligands (15) as well as an existence of a positive feedback loop, upregulating the expression of certain RAGE ligands (9, 16), suggest RAGE as a possible principal factor that promotes the development of certain immune mediated disorders, such as psoriasis. Moreover, RAGE is involved in the regulation of many proinflammatory genes those role in pathogenesis of psoriasis is already recognized (16, 17).

Aim of this paper is to describe the perspective pharmacological approaches that abolish the chronic inflammatory and immune responses *via* the inhibition of RAGE and suppression RAGE dependent signaling. Considering RAGE as a potential master regulator of several processes that play a crucial role in development of psoriatic plaques, such as secretion of proinflammatory cytokines (17, 18) and migration of T-cells to the sites of skin damage (13, 14) we believe that the development of novel medications that target RAGE and RAGE-dependent signaling mechanisms can be beneficial for all psoriasis patients.

## METHODS

Our objective was to systematically review the literature on different groups of drugs that interfere with RAGE and signaling cascades underlying this receptor. A systematic review of agents previously implicated in inhibition of RAGE and RAGE-dependent signaling pathways was undertaken, with a critical appraisal of the quality of the selected studies. This review focuses on the literature published from 2005 to December 2012. Three bibliographical databases (PubMed, Web of Science and MEDLINE) were used as data sources. The search results were supplemented by an extensive hand search of the literature through references identified from retrieved articles. The existing literature (*in vitro*, animal, and human studies) on this subject was considered.

Literature searches were performed in accordance with standardized procedures. The following searches were initially performed: “RAGE” AND “psoriasis”, “RAGE” AND “inhibitor”, “RAGE” AND “signaling”. The data were extracted by three independent researchers. The retrieved data were exchanged within this group and discussed on a joint meeting of five coworkers to resolve any discrepancies by consensus. The selected 5 major groups of agents were assigned to one of the co-authors for final evaluation and incorporation into the manuscript. The later, preceded the final editing of the text.

Thus, the searches identified 494 publications, of which 139 articles were included.

## RESULTS

### The extracellular matrix crosslinks breakers

Extracellular matrix crosslinks, which are among RAGE ligands, are resistant to enzymatic degradation (19). An elevation of AGEs levels is an important characteristic of the inflammatory response in psoriasis (20). Their interaction with RAGE stabilizes the receptor in the active state and initiates the production of proinflammatory cytokines (12, 15). However, certain agents can destroy AGE-derived protein crosslinks. The first identified AGE-crosslink breaker, PTB (N-phenacylthiazolium bromide), was introduced in 1996. Because only high concentrations of PTB (10-30 mM) can produce sufficient biological effects (21), this agent was not successful. Moreover, PTB is unstable *in vitro* (22). Later, several other compounds, such as ALT-711 (alagebrium chloride, phenyl-4,5-dimethylthiazolium bromide, Alteon Corporation/ Synvista Therapeutics, Inc, Montvale, NJ, USA) (23), LR20, LR23, LR90, (24, 25), metformin (Fortamet^®^, Watson Laboratories, Fort Lauderdale FL, USA /Glucophage^®^, Bristol-Myers Squibb, New York NY, USA /Glumetza^®^, Santarus, San Diego CA, USA /Riomet^®^, Ranbaxy Laboratories, Inc, Princeton NJ, USA) (26) and 3-[2-(4-bromo-phenyl)-1-methyl-2-oxo-ethyl]-4,5,6,7-tetrahydro-benzothiazol-3-ium bromide (27) were identified. Unlike PTB, these compounds produce the desired biological effect at lower concentrations (0.1-10 μM) and are more stable in aqueous solutions (21, 24, 26). The mechanism by which these compounds eliminate crosslinks is most likely similar to one that was described for PTB (28). Presumably, AGE-breakers cleave α-diketones by breaking the chemical bond between the carbonyl groups.

One of the best-studied crosslink breakers, ALT-711, exhibits encouraging results in mice. ALT-711 prevents the accumulation of AGEs in blood vessels (29, 30) and the heart (31, 32). Moreover, ALT-711 lowers the proliferation rate of neointimal cells, microvessel endothelial cells, and vascular smooth muscle cells (33). ALT-711 also decreases the phosphorylation of both PKC and Erk (33) and inhibits the AGE-dependent generation of ROS (31, 33) and expression of certain proinflammatory cytokines (31, 33). The expression of antioxidant enzymes, such as SOD and GSH-PX, is also decreased by treatment with ALT-711 (32). ALT-711 participated in several clinical studies for cardiovascular studies. Although the results of these studies suggest that ALT-711 is safe (34), they do not support ALT-711 benefits. Since 2007, clinical studies that involved ALT-711 are postponed due to financial problems.

To the date, the term “AGE-crosslink breakers” is not unanimously recognized due to certain inadequacy of *in vitro* and *in vivo* models (35). For instance, some authors argue of what could be a true transitional intermediate of the reaction (36). The others report that several attempts to confirm the results of *in vitro* modeling *in vivo* have failed (37, 38) and question the stability of AGE breakers in water solutions (22, 39). However, it is recognized that “AGE-crosslink breakers”, such as PTB and ALT-711 have a beneficial effect: these agents really delay AGEs accumulation *in vivo* (40). Several mechanisms, such as metal chelation (39-41), antioxidant activities (39, 40), and stimulation of the reductive pentosephosphate pathway (42) have been proposed to explain this biological effect. However, the recent data suggest that ALT-711 and related compounds indeed have the ability to cleave AGE-crosslinks. Kim and Spiegel report (43) that ALT-711 rescues *E. coli* from high concentrations of exogenously added methylglyoxal (MGO) by forming ALT-MGO adducts. Because the new data provide a support for the original model, in which ALT-711 functions *in vivo* as a selective mediator of C–C bond fragmentation (21) and clearly explain how ALT-711 might act therapeutically, we believe that time of “AGE-crosslink breakers” is yet to come.

### Non-conventional inhibitors of RAGE signaling pathways

***SAGEs. ***Among recently tested compounds for psoriasis treatment, there is a small group of sulfated anionic glycosaminoglycan ethers (SAGEs) that have the ability to inhibit AGEs. SAGEs are semi-synthetic oligo- and polysaccharides that are obtained by the modification of hyaluronic acid. These compounds should not be confused with the homonymous group of promitogenic and proinflammatory oligosaccharides, which are purified from the garden sage *Salvia officinalis L.* (44). One of the he SAGEs, GM1111, interferes with the binding of RAGE and its ligands (45). Moreover, this compound binds the antimicrobial peptide cathelicidin (LL37), which is a proposed mediator of inflammatory diseases, such as rosacea and psoriasis (46, 47). In turn, binding of SAGEs to LL37 suppress the induction of another inflammatory mediator S100A8 (45).

The main advantage of GM1111 before heparin that has a certain structural similarity with GM1111 is that GM1111 does not have sufficient anticoagulant activity. GM1111 also prevents the binding of leukocytes to P-selectin, impairing their ability to penetrate blood vessels (48). In addition, GM1111 inhibits the binding of human monoblastic/monocytic cell line U937 to RAGE *via* Mac-1 with an affinity that is comparable to heparin. Generally, the data suggest that SAGEs are potent proinflammatory agents and can be potentially used to elucidate the inflammatory response.

***KIOM-79, a natural RAGE inhibitor.*** Certain traditional pharmaceuticals that are used to treat psoriasis may eventually be replaced by the drugs that are obtained from natural sources. One of these products, KIOM-79, is developed in accordance with very old Eastern medicine traditions. KIOM-79 is a mixture of four components that are extracted from parched *Puerariae radix*, gingered *Magnoliae cortex*, *Glycyrrhizae rhizome*, and *Euphorbiae radix*. At the molecular level, KIOM-79 inhibits RAGE-dependent signaling mechanisms (49-51). KIOM-79 also activates AKT (52) and inhibits certain kinases, such as PKCA (49), ERK1/2 (51, 52) and p38 (49, 51, 53). In turn, the suppression of RAGE-dependent signaling pathways by this medication downregulates AP1- and NFκB-mediated transcription (52-56). At the transcriptional level, KIOM-79 treatment induces the expression of HO1 (52) and SOD (57), which are enzymes that destroy ROS. KIOM-79 also suppresses the expression of iNos (53, 56). However, this is not an exhaustive list of the biological effects of KIOM-79, which also suppresses VEGF (49, 58) and the accumulation of collagen (58). Unfortunately, KIOM-79 may not have a sufficient influence on cell proliferation (49). Thus, this anti-inflammatory medication may be used as a supplementary therapy.

### Inhibitors of intracellular signaling pathways

The discovery of RAGE and its role in the inflammatory response led some researchers to the idea that certain synthetic medications, such as ACE inhibitors (ACEI) that have anti-RAGE effects can be used on a broader spectrum of diseases, including psoriasis. The results of *in vitro* studies suggest that ACEIs slow down the accumulation of AGEs in cell cultures and also suppress cell proliferation (59-61). The *in vivo* studies have demonstrated that one of ACEIs, ramiprilat, stimulates secretion of the RAGE decoy form RAGE_v1. In turn, secretion of RAGE_v1 decreases soluble AGEs levels in blood as well as collagen-linked AGEs levels in the skin. Ramipril (Tritace^®^, Sanofi-Aventis, Paris, France/Altace^®^, King Pharmaceuticals, Bristol, TN, USA) and at least three other ACEIs exhibit certain important similarities in their biological effects. The administration of perindopril (Aceon^®^, Solvay Pharmaceuticals, Marietta, GA, USA) increases the blood concentrations of soluble RAGE in patients with hyperglycemia (62). Benazepril (Lotensin^®^, Novartis Basel, Swiss) sufficiently suppresses both the accumulation of AGEs and RAGE expression. Moreover, benazepril inhibits RAGE-dependent signaling mechanisms, suppressing the expression of NFκB p65, p-NFκB p65, VCAM1 and TGFβ1 NADP oxidase, as well as the formation of ROS (63-65). In turn, captopril slows down the accumulation of collagen type I and inhibits JAK2-STAT1/STAT3 signaling (66). Thus, the strong antiproliferative effect of ACEIs as well as the strong anti-RAGE effect suggest this group of medicines as a potential treatment of psoriasis.

In human patients with psoriasis, the medical effect of ACEIs may vary. In certain clinical cases, captopril improved the symptoms of the disease (67); in many other studies, however, this medication made psoriasis worse (68-72). Similar results have been observed for enalapril (Vasotec^®^, Valeant Pharmaceuticals International, Laval QC, Canada/Enaladex^®^, Dexcel Pharma, Jerusalem, Israel) (73) and ramipril (74). Moreover, two different ACEIs, captopril (Capoten^®^, Bristol-Myers Squibb, New York NY, USA) and perindopril, may have opposite effects on the same patient (75). The later suggests that ACEIs are likely to have several molecular targets and their interplay may rather worsen psoriasis than interfere with general course of the disease. Generally, the published cases suggest that patients with psoriasis should use ACEIs very cautiously. Hopefully, the effects of these drugs on psoriasis will be clarified in future studies.

In the context of psoriasis, protein tyrosine kinases (PTK) may 1) affect the transmission of signals from RAGE to NFκB, 2) contribute to activation of keratinocytes and immune cells *via* their participation in induction of growth factors genes, and 3) also participate in angiogenesis. The published data suggest that selective inhibitors of protein tyrosine kinases may be of commercial interest. Unsurprisingly, the RAF kinase, EGFR and VEGFR inhibitors that are targeting the key molecules of these three processes are already marketed in connection with other diseases. These drugs are widely available given that the majority of the developed tyrphostins (inhibitors of tyrosine phosphorylation) were developed as treatments for hyperproliferative diseases, such as metastatic cancer. Moreover, tyrphostins can also be helpful in treating psoriasis, although the majority of the observations regarding these compounds come from their use in treating patients with advanced forms of cancer.

Approximately 15 years ago, SU 5271 (sunitinib, Sutent^®^, Pfizer. New York NY, USA) was the only tyrphostin that was being tested in clinical trials (76). Presently, the situation has changed. In 2006, the FDA approved sunitinib for the treatment of renal cell carcinoma (RCC) and for certain categories of gastrointestinal stromal tumors (GIST). The ability of sunitinib to inhibit multiple tyrosine kinase receptors, such as PDGFR, VEGFR (77, 78), KIT (77), RET (79), CSF1R, RAF and FLT3 (77) creates a risk for worsening numerous skin conditions [discussed in (80)]. However, sunitinib has been occasionally used to control severe psoriasis and even as a substitute drug following the eruption of the disease (81, 82).

Unfortunately, broad specificity of tyrphostins that are already available on the market represents a certain risk rather be beneficial. For example, sorafenib, which inhibits VEGFR, PDGFR and RAF1, may actually promote cancer growth (83, 84). Sorafenib monotherapy can also lead to other cutaneous reactions, such as facial erythema, scalp dysesthesia, and alopecia (85, 86), as well as the inflammation of actinic keratoses (83, 87, 88). The reported cases of patients who suffer from both cancer and psoriasis suggest that a history of skin cancer should be taken in consideration before the patient is prescribed sorafenib and suggest that lower dosages should be used for their treatment (89).

Another tyrphostin, imatinib (Gleevec^®^/Glivec^®^, Novartis, Basel, Swiss), inhibits Abelson tyrosine kinase (ABL1), c-Kit, PDGFR, c-FMS and ZAP70 (zeta-chain-associated protein kinase 70). This drug may cause dermatologic toxicity, and its most common associated adverse events are maculopapular eruptions, erythematous eruptions, edema, and periorbital edema [reviewed in (90)]. Although imatinib may improve psoriasis in certain patients (91), it is necessary to acknowledge that the simultaneous inhibition of several signaling pathways is a very risky and unjustified approach. There are several reported cases of a psoriasis eruption following imatinib treatment (90, 92, 93). Moreover, the lab tests that were performed on one of the reported cases suggest a link between imatinib and increased numbers of Th1 cells, which are a part of the psoriatic infiltrate (94).

Tyrphostins that specifically target EGFR may also lead to improvements of skin conditions in patients with psoriasis (95-97). However, prescribing medication that targets EGFR may, unsurprisingly, cause unexpected results due to the crucial role of EGF in the terminal differentiation of keratinocytes. This effect has been confirmed by numerous clinical cases in which dermatological toxicity, including psoriatic lesions, was observed as a side effect (98-100). Their brief analysis suggests that, skin cancer and leukemia are major risk factors for using EGFR inhibitors in patients predisposed to psoriasis (81, 89, 90, 94, 101, 102).

Despite past failures, drugs that target certain intracellular pathways will likely become competitive products on the market once again. The successors of previously used medications must be more selective on their targets. This new group of medicines has a high probability of becoming a new cost-effective alternative to certain existing treatments. Particularly, JAK kinases can be considered to be good examples of such drugs. The choice of JAK kinases as molecular targets for autoimmune diseases is based on their role in the activation, differentiation and functioning of immune cells, primarily T-cells. One of the JAK kinases, JAK3, is the only known mediator of the type I cytokine receptor with the common γ-chain (γc) (103). Downstream JAK3 signaling is linked to the expression of proinflammatory cytokines and other genes that are involved in the activation, proliferation and selection of T-cells (104-107). In humans, JAK3 deficiency is associated with severe combined immunodeficiency (SCID), which is characterized by profound defects in T- and NK-cell development (108). In mice, the genetic ablation of Jak3 (109) causes dramatic defects in development of T-, B- and NK-cells. Jak3 -/- mice display T and B cell lymphopenia without effects on myeloid lineage cells (i.e., Gr-1 positive granulocytes, erythroid cells and Mac-1 monocytes) (110, 111). In contrast, abnormal JAK3 activation is associated with acute megakaryoblastic leukemia (AMKL) and cutaneous T cell lymphoma (CTCL) (112, 113). Because the JAK3-mediated step of the signaling cascade is critical for immune cell development and differentiation, its targeting may be beneficial for the treatment of many autoimmune conditions, such as psoriasis. To date, a large group of inhibitors of JAK3 is under development.

***AG-490.*** AG-490 is a membrane-soluble tyrosine kinase inhibitor. This compound was likely one of the first drugs that exhibited a proven capacity to inhibit JAKs, primarily JAK2. Thus, AG-490 suppresses several signaling pathways, including IL-2-mediated signaling, which is upstream of JAK3 and STAT5A and B. Generally, AG-490 can be used to control the abnormal constitutive activation of JAK2 in leukemia cell lines (114).

***CP-690,550.*** CP-690,550 (tofacitinib /tasocitinib, Xeljanz^®^, Pfizer, New York NY, USA, is an orally administered inhibitor of JAK3 that was initially developed by Pfizer as an immunosuppressive drug for use in transplant patients (115). Despite inhibiting JAK3, the affinity of CP-690,550 for JAK1 is even greater (116).

Experimental studies confirm the immunosuppressive potential of CP-690,550. *In vitro*, CP-690,550 blocks the differentiation of Th cells (117). Particularly, it inhibits IL-4-dependent Th2 cell differentiation (118). Moreover, as an inhibitor of JAK1 and JAK3, CP-690,550 also decreases signaling through STAT1 and Th1 cell-specific transcription factor, T-bet, that controls IFNG expression. In turn, IFNG suppression reduces the expression of chemokines and cytotoxic effector molecules (e.g., IP-10, RANTES and MCP1) (119). According clinical study results, CP-690,550 does not cause any sufficient changes in the number of neutrophils, total lymphocytes, platelets, CD4^+^ and CD8^+^ T cells. Contrary, treatment with CP-690,550 decreases NK cell counts by 50% and increases the CD19^+^ B-lymphocytes count (120). Although this effects are reversible they occur even at low doses.

Pfizer proposes CP-690,550 for the treatment of many diseases including psoriasis (121). The first clinical trial to use this compound to treat psoriasis has been conducted in 2008 (122). The report reveals that at day #14 of the study, the clinical score PLSS (Psoriatic Lesion Severity Sum score) drops in 25-75% of the patients in a dose-dependent manner, and certain patients exhibit complete remission. The side effects CP-690,550 include headache, nausea and elevated levels of both low-density lipoprotein (LDL) and high-density lipoprotein (HDL). Moreover, respiratory infections are more common in the patients who receive CP-690,550 *vs*. the placebo group. CP-690,550 is now in phase II clinical trials. Moreover, in September 2010, Pfizer initiated several phase III clinical trials to assess the efficacy of oral tofacitinib in patients with moderate to severe chronic plaque psoriasis.

***Dimethoxyquinazoline compounds.*** Originally, dimethoxyquinazolines were developed to induce apoptosis in leukemic cells (123). Because JAK3 is overexpressed in leukemia, researchers have sought to identify a drug that was capable of fitting into the JAK3 catalytic domain (124). Notably, dimethoxyquinazolines are non-toxic in mice and in non-human primates (125). These compounds have a very high clearance and relatively low IC50 values, i.e., between 0.1 and 100 µM. In addition to JAK3, dimethoxyquinazolines also inhibit JAK1 and JAK2, although with a lower efficiency than their inhibition of JAK3 (126). They also inhibit certain other non-JAK kinases, such as ABL, LCK, SRC, and VEGFR. Because dimethoxyquinazolines exhibit high cross-reactivity with kinases of the EGF receptor family (124), their broad specificity may explain serious adverse effects of these drugs, such as disrupted platelet functions.

***NC1153.*** NC1153 was discovered among 200,000 synthetic, semi-synthetic and natural products in the National Cancer Institute’s drug discovery program (127). This agent is a Mannich base (β-amino-ketone). NC1153 is an irreversible inhibitor of JAK3 (128), and it is 40 times more selective for JAK3 than for JAK2. In rats, NC1153 prevents the phosphorylation of Stat 5a and b and Erk1/2 (127). In the same time, this agents is well-tolerated by rats and does not exhibit myelo-, lipo- or nephrotoxicity in lab animals (127-129).

***PF-956980.*** Since the development of CP-690,550, Pfizer has developed several other drugs with improved selectivity. One of these new compounds, PF-956980, is structurally related to CP-690,550. Comparative studies of JAK3 inhibitors have confirmed that PF-956980 has a higher affinity to JAK3 than CP-690,550 and more selective to JAK3 than AG490, WHI-P131 and WHI-P154 in an array of 30 different protein kinases (124).

***PNU156804. ***PNU156804 (Pfizer, New York, NY, USA) is an antibiotic of the undecylprodigiosin family and is isolated from *Serratia marcescence*. This agent attracted the attention of researchers for its ability to block IL2-induced T-cell proliferation (130). The observed effects are limited to cells that already express JAK3, and its action is not limited to STAT5A and STAT5B. In psoriasis, keratinocytes, which also express JAK3 are among such target cells. PNU156804 also suppresses two other important transcription factors: AP1 and NFκB. Currently, the development of PNU156804 has ceased due to its high myelosuppressive toxicity (127).

***R348.*** R348 (Rigel Pharmaceuticals, San Francisco CA, USA) is a pro-drug that was developed by Rigel Pharmaceuticals as a JAK3-selective inhibitor that also inhibits spleen tyrosine kinase (SYK). Preclinical studies of R348 suggest its selectivity for JAK3 over JAK2 in cellular assays (131-133). In CD18-deficient mice with a psoriasis-like phenotype, the active metabolite of R348, R333 (Rigel Pharmaceuticals, San Francisco CA, USA), inhibits Jak3- and Syk-dependent pathways and reduces cellular Th1 and Th2 immune responses (131). Treatment with R348 also results in higher levels of alanine transferase in the blood. However, R348 does not induce hypercholesterolemia and reduces systemic levels of IL17, IL22, IL23, and TNF, which are important for controlling psoriasis.

***Ruxolitinib.*** Ruxolitinib (INCB018424, Jakafi^®^) is developed by Incyte Corp. and Novartis AG (134). This is a potent JAK1 and JAK2 inhibitor that is currently under investigation for the treatment of psoriasis (135). As a dual JAK1 and JAK2 inhibitor, this medicine has the potential to modulate signal transduction from a variety of cytokines implicated in the pathogenesis of psoriasis, including interleukin IL12 and IL23 (135, 136). Ruxolitinib is also an investigational compound for some other diseases such as myeloproliferative neoplasm and polycythemia vera. In preclinical studies, ruxolitinib reduced the pro-inflammatory cytokine signaling mediated by JAK1 and JAK2 in lymphocytes and monocytes, with half-maximal inhibitory concentration values <100 nM (135, 137). *In vivo*, topical application of ruxolitinib results in suppression of STAT3 phosphorylation. This agent also abolishes edema, lymphocyte infiltration, and keratinocyte hyperproliferation in a murine contact hypersensitivity model. As an adverse effect, ruxolitinib may cause low blood cell counts (white blood cells, red blood cells and platelets), which can be reversed by stopping ruxolitinib or reducing the dose (138).

***VX-509.*** VX-509 (Vertex Pharmaceuticals, Cambridge, MS, USA) is a selective JAK3 inhibitor that is developed by Vertex Pharmaceuticals. Currently, it is in a phase II clinical trial for rheumatoid arthritis (NCT01052194). However, the lack of published clinical trial data precludes any proper assessment of its selectivity or effectiveness.

***WYE-151650.*** WYE-151650 has been confirmed to inhibit IL2-induced STAT5 phosphorylation *in vitro*. This agent also exhibits a high selectivity to JAK3 compared to JAK1, JAK2, and TYK2, (36-, 14-, and 34-fold, respectively) and more than 100-fold selectivity over 27 non-JAK family kinases. Pharmacokinetic studies suggest a good absorption of WYE-151650. Moreover, this agent has 36% oral bioavailability in mice. *Ex vivo*, WYE-151650 suppresses IL-2-induced STAT5 phosphorylation in blood cells and inhibits the proliferation of peripheral blood mononuclear cells. *In vivo*, WYE-151650 inhibits the JAK-3-mediated production of IFNG and has the same potency as CP-690,550. In addition, WYE-151650 decreases the natural killer cell population in mice, and its effect on NK cells is comparable to that of CP-690,550 (139).

## CONCLUSION

In this manuscript, we discuss different pharmacological approaches that can be used for treatment of psoriasis. Considering that the development of psoriasis is a complex, multistep process that involves different cell types and signaling pathways, we presume that psoriasis can be controlled *via* the inhibition of certain signaling molecules such as receptors and kinases. A growing number of clinical studies that involve JAK inhibitors speaks in favor of this opinion. Taking in account high cost of biologics, which are the most efficient treatment of medium and severe forms of psoriasis, we propose a wider use of other medicines, such as new commercial inhibitors of signaling molecules. These new agents will be more selective on their targets and have a higher affinity. They will also have fewer side effects. Thus, we strongly believe that this publication will be helpful attract attention of readers those goal is to propose new solutions for the existing long-term medical problems and who are involved in a discovery of new medicines.

## ABBREVIATIONS

ABL1: Abelson tyrosine kinase;
ACE: angiotensin converting enzyme;
AGE: advanced glycation end-products;
ALT-711: alagebrium chloride;
AMKL: acute megakaryoblastic leukemia;
CTCL: cutaneous T cell lymphoma;
GIST: gastrointestinal stromal tumors;
GSH-PX: Glutathione peroxidase;
HDL: high-density lipoprotein;
JAK: Janus kinases;
LDL: low-density lipoprotein;
LL37: cathelicidin;
MGO: methylglyoxal;
PTB: N-phenacylthiazolium bromide;
PTK: protein tyrosine kinases;
RAGE: receptor for advanced glycation end-products;
RCC: renal cell carcinoma;
ROS: reactive oxygen species;
SAGE: sulfated anionic glycosaminoglycan ethers;
SCID: severe combined immunodeficiency;
SOD: superoxide dismutase;
ZAP70: zeta-chain-associated protein kinase 70;
